# Diffuse reflectance spectroscopy accurately quantifies various degrees of liver steatosis in murine models of fatty liver disease

**DOI:** 10.1186/s12967-015-0671-1

**Published:** 2015-09-21

**Authors:** Andrie C. Westerkamp, Vishnu V. Pully, Golnar Karimian, Fernanda Bomfati, Zwanida J. Veldhuis, Janneke Wiersema-Buist, Benno H. W. Hendriks, Ton Lisman, Robert J. Porte

**Affiliations:** Section of Hepatobiliary Surgery and Liver Transplantation, Department of Surgery, University of Groningen, University Medical Center Groningen, P.O. Box 30.001, 9700 RB Groningen, The Netherlands; Surgical Research Laboratory, Department of Surgery, University of Groningen, University Medical Center Groningen, Groningen, The Netherlands; In-Body Systems Department, Philips Research, Eindhoven, The Netherlands

## Abstract

**Background:**

A real-time objective evaluation for the extent of liver steatosis during liver transplantation is currently not available. Diffuse reflectance spectroscopy (DRS) rapidly and accurately assesses the extent of steatosis in human livers with mild steatosis. However, it is yet unknown whether DRS accurately quantifies moderate/severe steatosis and is able to distinguish between micro- and macrovesicular steatosis.

**Methods:**

C57BL/6JolaHsd mice were fed wit a choline-deficient l-amino acid-defined diet (CD-AA) or a choline-sufficient l-amino acid-defined control diet (CS-AA) for 3, 8, and 20 weeks. In addition B6.V-*Lepob*/OlaHsd (ob/ob) mice and their lean controls were studied. A total of 104 DRS measurements were performed in liver tissue ex vivo. The degree of steatosis was quantified from the DRS data and compared with histopathological analysis.

**Results:**

When assessed by histology, livers of mice fed with a CD-AA and CS-AA diet displayed macrovesicular steatosis (range 0–74 %), ob/ob mice revealed only microvesicular steatosis (range 75–80 %), and their lean controls showed no steatosis. The quantification of steatosis by DRS correlated well with pathology (correlation of 0.76 in CD-AA/CS-AA fed mice and a correlation of 0.75 in ob/ob mice). DRS spectra did not distinguish between micro- and macrovesicular steatosis. In samples from CD-AA/CS-AA fed mice, the DRS was able to distinguish between mild and moderate/severe steatosis with a sensitivity and specificity of 86 and 81 %, respectively.

**Conclusion:**

DRS can quantify steatosis with good agreement to histopathological analysis. DRS may be useful for real-time objective evaluation of liver steatosis during liver transplantation, especially to differentiate between mild and moderate/severe steatosis.

## Background

A widely adopted strategy to increase the number of liver donors is the use of extended criteria donor (ECD) livers [1]. One of the most prevalent conditions to classify a graft as ECD liver is hepatic steatosis. According to histopathological evaluation, steatosis can be categorized in a qualitative and quantitative manner. Qualitatively, steatosis is subdivided into two different histological patterns: micro- and macrovesicular steatosis. Quantitatively, steatosis is classified according to the percentage of hepatocytes affected by fat vacuoles: mild (less than 30 %), moderate (between 30 and 60 %), or severe (above 60 %) [2–4]. How steatosis affects graft survival depends on the type and degree. Donor livers with microvesicular steatosis (in all degrees) and with mild macrovesicular steatosis are suitable for transplantation. On the other hand, livers with moderate and severe macrovesicular steatosis are in most cases considered as a contraindication for transplantation due to their relation with poor postoperative outcome [2–4].

Given the rising incidence of obesity, the general expectation is that more steatotic livers will become available for liver transplantation. However, evaluation of the degree and type of steatosis is still a challenge for the surgical team during organ procurement procedures. Currently, assessment of hepatic steatosis requires a liver biopsy. However, processing and examination of the biopsy is a logistic challenge during off-hours, when most organ procurement procedures take place. Moreover, tools for a quick and accurate assessment of the type and degree of steatosis are not yet available [4].

Recently, diffuse reflectance spectroscopy (DRS) has demonstrated its possibilities to measure lipid concentration in tissue minimal invasively, accurately and in real-time [5–9]. In a recent study performed by our group, DRS was able to quantify only mild degrees of steatosis [10]. However, from a clinical perspective, it is also necessary to determine if the DRS is able to discriminate between mild and moderate/severe macrovesicular steatosis. In addition, the ability of the DRS to discriminate between micro- and macrovesicular steatosis is yet unknown.

The aim of the current study is therefore to investigate if DRS could quantify moderate and severe steatosis and if DRS could differentiate between micro- and macrovesicular steatosis. Because (discarded) human donor livers with mild and moderate steatosis are not available in large quantities, we used two well-established mouse models (choline-deficient diet and leptin deficiency; ob/ob) of hepatic steatosis.

## Methods

### Animals

C57BL/6JolaHsd mice (5–6 weeks old) and B6.V-*Lepob*/OlaHsd (ob/ob) mice (9–10 weeks old) were purchased from Harlan Laboratories (Boxmeer, the Netherlands). Animals were caged in animal rooms with an alternating 12-h light/dark period and had free access to food and water for the duration of the experiment. All procedures were approved by and performed in compliance with the Institutional Animal Care and Use Committee of the University of Groningen (IACUC-RuG).

### Experimental design

**Fig. 1:**
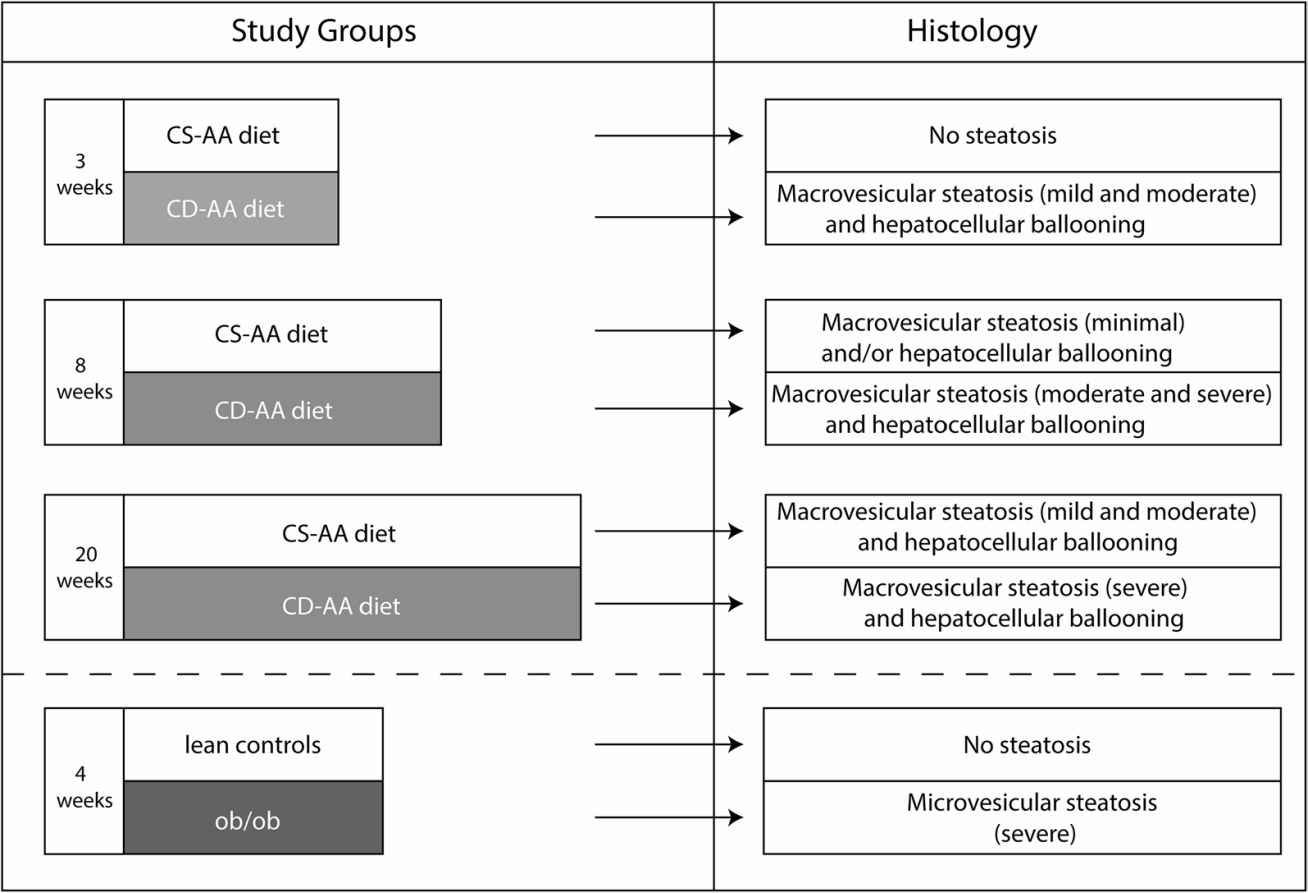
Overview of the experimental groups and type and grade of hepatic fat content. To induce hepatic steatosis, mice received the choline-deficient l-amino acid-defined (CD-AA) diet for 3, 8, and 20 weeks. The control mice were fed with choline-sufficient l-amino acid defined (CS-AA) chow diet for the same time periods. The ob/ob (leptin deficient) mice and their lean controls received the standard chow diet for 4 weeks. After the specific weeks of diet, DRS measurements were performed in liver tissue ex vivo

To induce different stages of hepatic steatosis, the C57BL/6JolaHsd mice received a choline-deficient l-amino acid-defined (CD-AA) chow diet (no. 518753, Dyets Inc., PA, USA) for 3, 8, and 20 weeks. The control mice were fed with a choline-sufficient l-amino acid defined (CS-AA) chow diet (no. 518754, Dyets Inc., PA, USA) for the same time periods. The ob/ob mice and their lean controls received a standard chow diet (reference no. 2181 AB Diets, Woerden, the Netherlands) for 4 weeks (Fig. 1). Each group comprised of 5–7 mice, for a total of 52 mice.

**Fig. 2:**
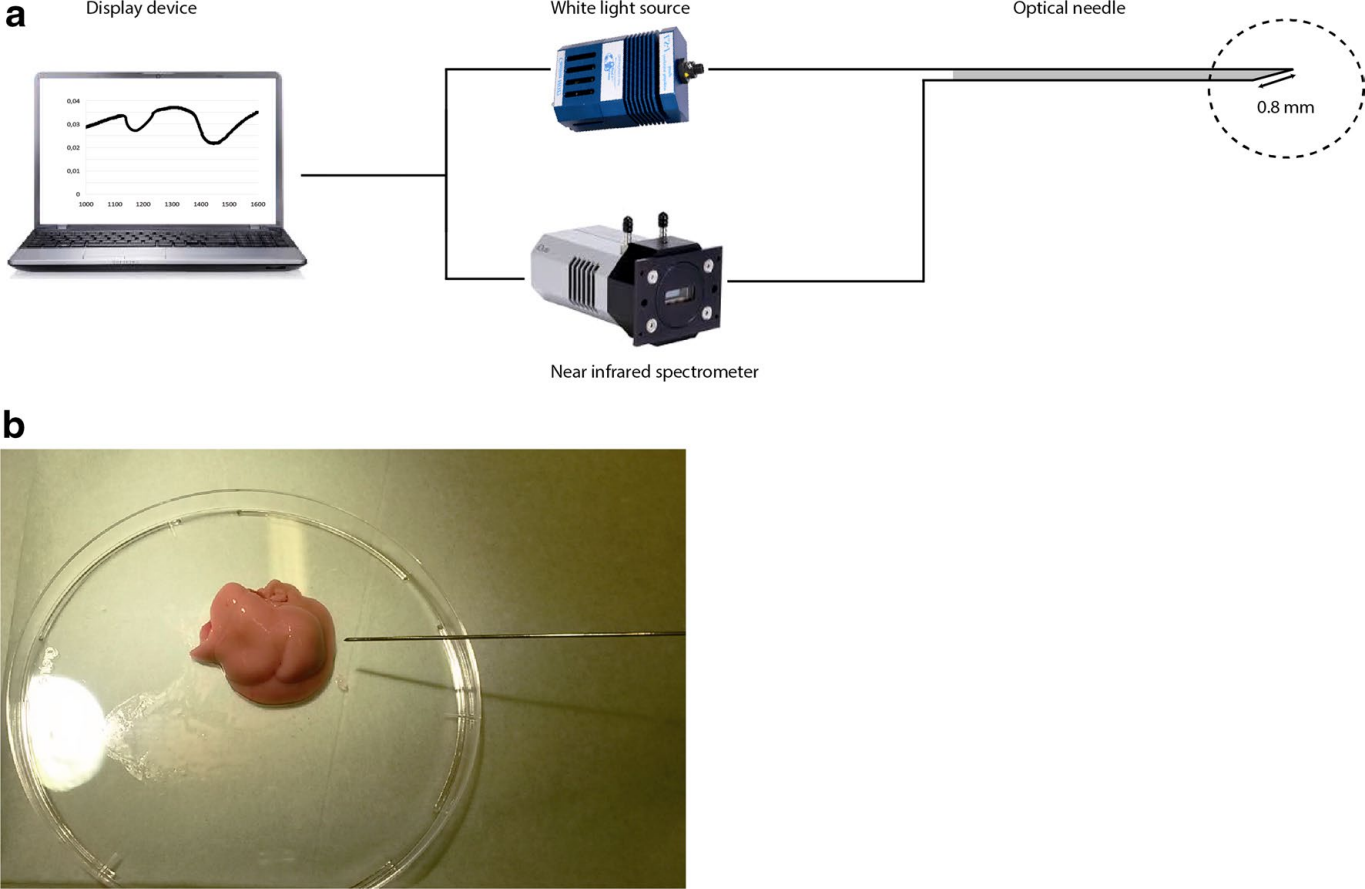
Overview of the DRS system and optical measurement. *Panel* **a** set-up of the DRS system with the optical needle. The diameter of the needle is 0.91 mm (20 Gauge) with a fiber distance of 0.8 mm. *Panel* **b** ex vivo measurement in mouse liver tissue with DRS needle

After 3, 4, 8 or 20 weeks of feeding, mice were anesthetized using a mixture of isoflurane/O_2_, subjected to a hepatectomy, and sacrificed. After the hepatectomy, the liver was stored on a petri dish for immediate measurement with the DRS (Fig. 2). Per mouse liver, two liver lobes (left median and right upper) were used for ex vivo measurements with the optical needle of the DRS system. The optical needle was inserted once in each liver lobe. Per lobe, five consecutive DRS measurements were performed, which were averaged. Thereafter, a biopsy was taken from the specific measurement location for further histopathological analysis.

### Optical spectroscopy instrumentation and analysis

Recently, the instrumentation and calibration procedure of the DRS system was described by Nachabé et al. [6]. In brief, the DRS system consists of a console comprising a broadband light source and one spectrometer (Fig. 2). The spectrometer resolve light in the near infrared wavelength range from 1000 up to 1600 nm. Within the optical needle one fiber was connected to the light source and one fiber was connected to the spectrometer to capture the diffusely scattered light from the tissue. The diameter of the needle is 20 Gauge (0.91 mm) with a fiber distance of 0.8 mm. The average tissue volume that is illuminated with the needle is roughly 1 mm^3^. The acquisition time of each spectrum was on average 0.2 s.

Analysis of the spectral data has been described previously [10]. In brief, each cellular component such as lipid, hemoglobin or collagen (component of fibrosis) has its own intrinsic optical characteristic (spectrum), which consists of a combination of absorption and scattering coefficients in a specific wavelength range. From our earlier experiments, the spectra of lipid, hemoglobin and collagen are identified. Fat and collagen are the dominant chromophores in the wavelength range between 1100 and 1600 nm, while (oxygenated and deoxygenated) hemoglobin have the dominant chromophores in the wavelength range of 500–900 nm [11]. Moreover, the absorption coefficients of lipid compared to collagen are different [11]. Dependent of the spectra and combination of absorption and scattering coefficients, the DRS is able to estimate the hepatic lipid (fat) content. In addition, scattering in the wavelength range 1000–1600 nm is dominated by Mie scattering. The reduced scattering coefficient μ_s_′(λ) is modeled by μ_s_′(λ) = α λ^−b^ where λ is the wavelength and b the Mie-slope [11]. Furthermore, it is known that contact pressure of the needle affects the absorption, scattering and intensity of the spectra [12–15], we performed measurements very thoroughly in order to apply similar contact pressure on each liver specimen. Spectral characteristics analysis (fitting of the data) was performed with a Matlab software package (MathWorks Inc., Natick, MA, USA).

### Histological analysis

Biopsy specimens from the optical measurement locations were first fixed in formalin, then paraffin embedded and subjected to standard hematoxylin and eosin (H&E) staining. For assessment of macrovesicular steatosis, the sections were digitally scanned using a digital whole slide scanner (Aperio ScanScope GS, Aperio Technologies, Vista, CA, USA) at a magnification of 40 (100,000 pixels/inch). Per section, eight digital images were saved as TIFF files. Next, a computer-based image-processing algorithm was developed in ImageJ (National Institutes of Health, Bethesda, MD, USA) for morphometric calculations of the macrovesicular lipid droplet area, as described previously [7]. The lipid droplets were identified by their shape and diameter. Non-hepatocyte areas such as sinusoids, vessels and artifacts (tissue cracks) were excluded. Macrovesicular steatosis was enumerated for each picture as the percentage of surface consisting of lipid droplets per total surface area. The morphometric calculations with ImageJ were not applicable for microvesicular steatosis and hepatocellular ballooning. Assessment of microvesicular steatosis and hepatocellular ballooning was therefore performed visually with light microscopy. Microvesicular steatosis was defined as innumerable tiny lipid vesicles that were diffusely distributed and causes a foamy appearance of the cytoplasm. In hepatocellular ballooning, no cytoplasmic fat vacuoles are present; hepatocytes are swollen due to lipid accumulation in nonvesiculated areas [16]. Scoring of microvesicular steatosis and ballooning was performed in a blind fashion by two independent observers (ACW and FB) according to the established scoring method of Kleiner et al. [17]. The lipid fraction scored by pathological evaluation was considered to be a two dimensional analysis of the same three dimensional volume of liver tissue analyzed with DRS. To adjust the pathological scored lipid fraction to a volume lipid fraction, the pathological lipid fractions were recalculated using the principle postulated by Weibel et al. [18]. Moreover, it should be noticed that between histological scoring and DRS analysis an intrinsic difference of 5 % is obtained due to that lean mice livers without steatosis also contain some intracellular lipids [19]. In order to score DRS analysis as positive, the determined fat fraction should be above the threshold value of 5 %.

### Statistical analysis

Spearman’s rank correlation test was used to examine the correlation between the DRS ex vivo measurements and the histological quantification of steatosis. Linear regression (r^2^) was used to assess correlations between DRS measurements and histological quantification of steatosis. The statistical differences between the scattering parameters were determined using a non-parametric Kruskal–Wallis test [20]. P levels smaller than 0.05 were considered as statistically significant. Analyses were performed using GraphPad Prism version 5.00 for Windows (GraphPad Software Inc., La Jolla, CA, USA).

## Results

### Histological assessment

Histological evaluation of the liver biopsies of mice in the CD-AA diet group showed macrovesicular steatosis in the range from 17 to 73 %. The degree of macrovesicular steatosis increased with the duration of the diet. In addition, ballooned hepatocytes were observed in the CD-AA diet group with an increased number together with an increasing duration of the diet. Mice fed with CS-AA diet for 3 weeks displayed no steatosis in their biopsies. However, 8 and 20 weeks of CS-AA diet also resulted in the development of macrovesicular steatosis and/or hepatocellular ballooning but these degrees were lower than the CD-AA diet group. The ob/ob mice developed exclusively microvesicular steatosis, which was severe (range 75–80 %) in all animals. Evaluation of the liver biopsies of their lean controls showed no evidence of hepatic fat infiltration (Fig. 1).

**Fig. 3:**
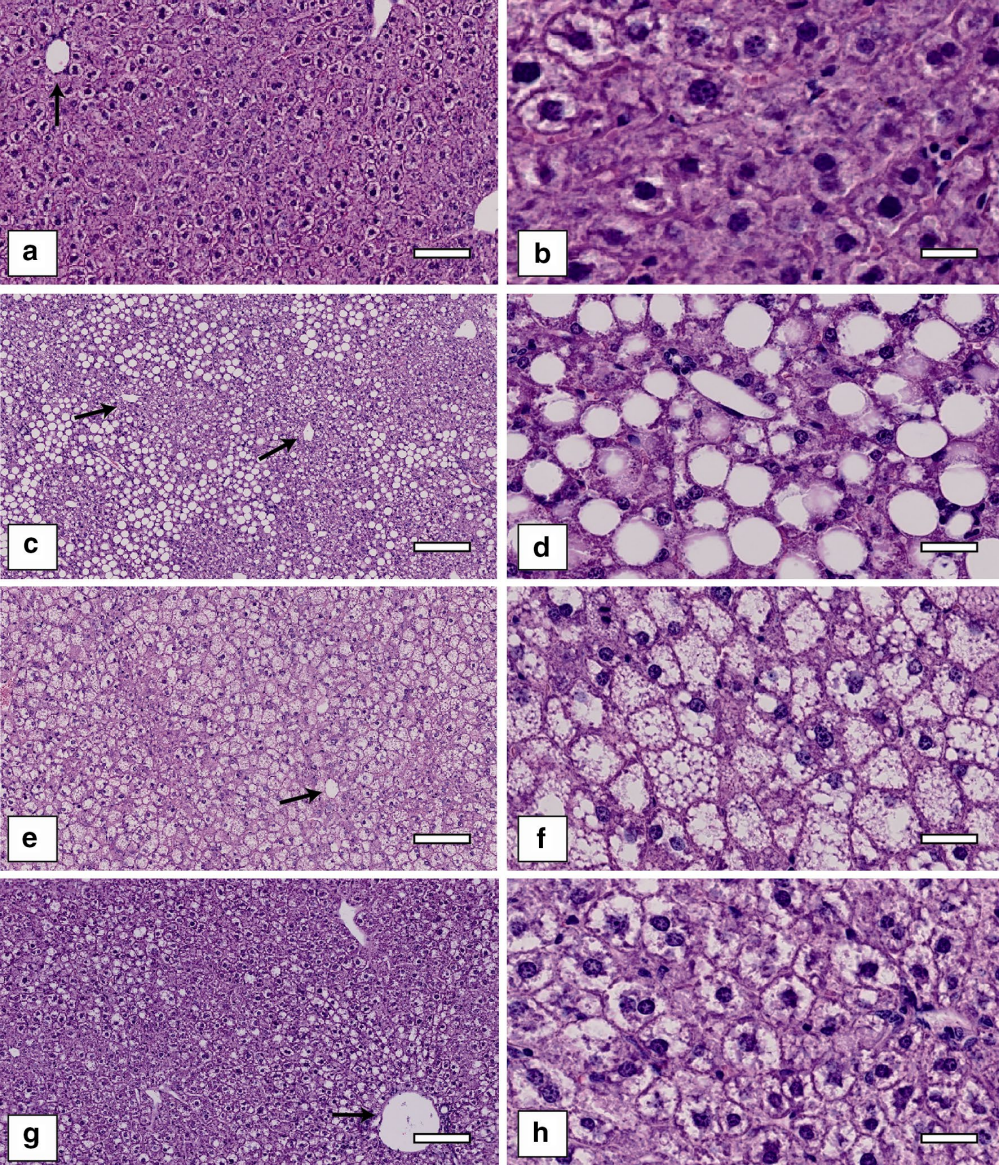
Overview of normal hepatic mouse tissue and different types of hepatic fat filtration. *Panels* **a**, **b** normal hepatic mouse tissue, *panel* **b** is a magnification of *panel* **a**. *Panel* **c**, **d** macrovesicular steatosis, *panel* **d** is a magnification of *panel* **c**. *Panels* **e**, **f** microvesicular steatosis, *panel* **f** is a magnification of *panel* **e**. *Panels* **g**, **h** hepatocellular ballooning, *panel* **h** is a magnification of *panel* **g**. *Black arrows* point to the central vein. All sections are H&E stained. *Scale bars* in the *panels* **a**, **c**, **e**, and **f** indicate 500 μm. *Scale bars* in the *panels* **b**, **d**, **f**, and **h** indicate 50 μm

**Fig. 4:**
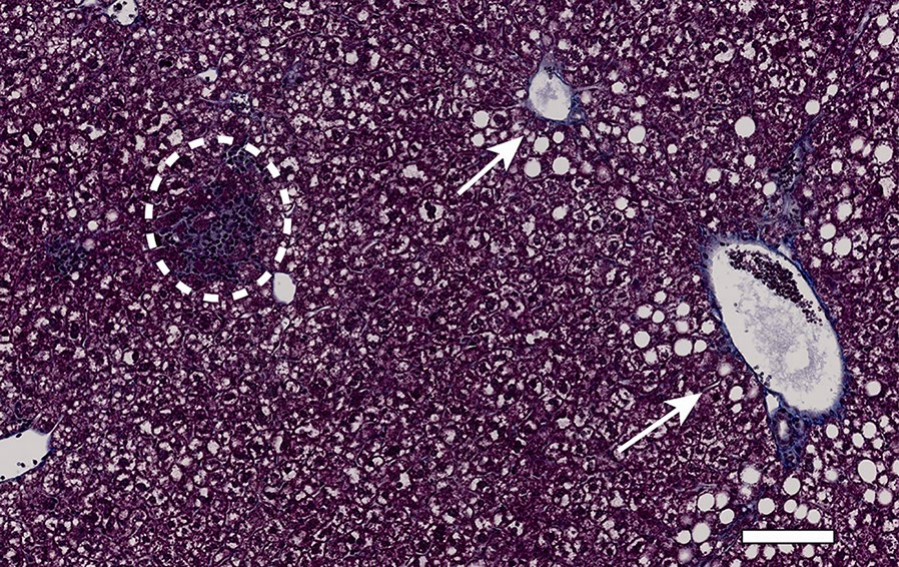
Masson trichrome staining of mouse liver in the CD-AA diet group. Clusters of inflammatory cells are visualized in the *dashed white circle*. *White arrows* show fibrosis (periportal and perivenular). *Scale bar* indicates 100 μm

Figure 3 shows examples of the different types of hepatic fat infiltration. The large fat droplets of macrovesicular steatosis were predominately present in clusters around the central vein. In particular, macrovesicular steatosis was not evenly distributed throughout the liver. The patterns of microvesicular steatosis as well as hepatocellular ballooning were more heterogeneous distributed in the liver parenchyma. In addition, Fig. 4 illustrates an example of liver biopsy with macrovesicular steatosis, ballooning, inflammation, and periportal/perivenular fibrosis, which were mainly observed in the late stages of the CS-AA and CD-AA diet.

### Evaluation of steatosis with DRS

**Fig. 5:**
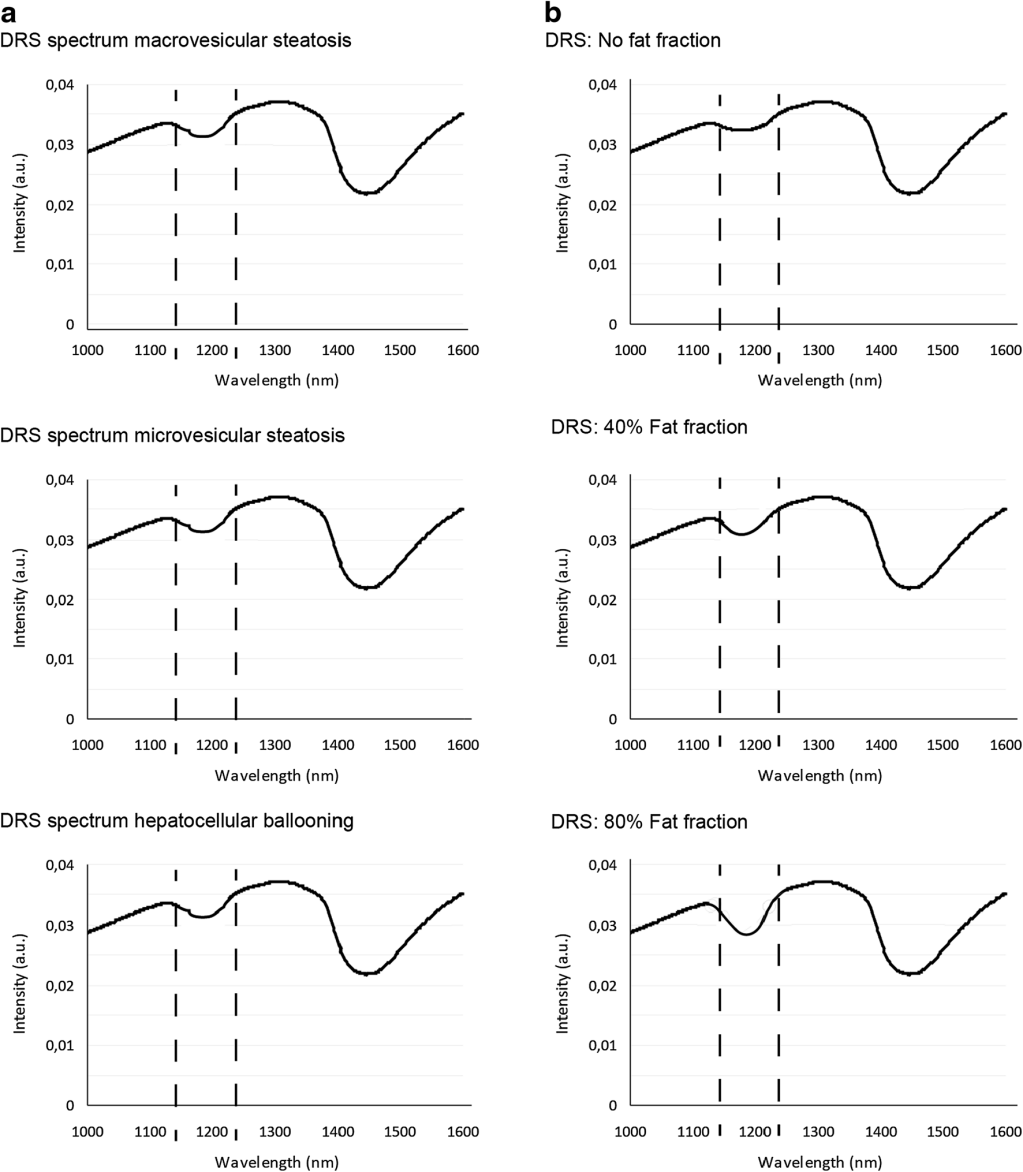
Examples of spectra measured with the DRS in different types of hepatic fat infiltration and various degrees of steatosis. *Panel* **a** the *dashed lines* correspond to the wavelength section (around 1200 nm) for which the spectra are altered by the presence of lipid. *Panel* **b** examples of spectra of normal hepatic mouse tissue and fatty mice livers with an increasing severity of macrovesicular steatosis. A more prominent inverse peak in the light spectrum in the vicinity of 1200 nm is related to a higher fat concentration in the measured tissue. *A.u.* indicates arbitrary units

**Fig. 6:**
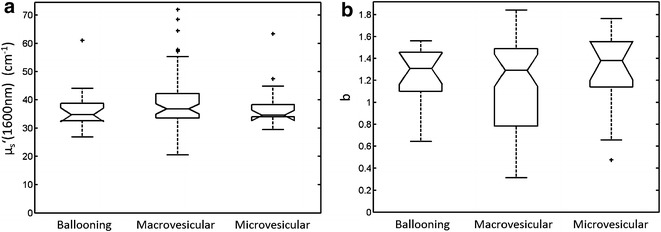
*Boxplots* of the reduced scattering coefficient at 1600 nm and the corresponding Mie-slope “b” for micro- and macrovesicular steatosis, and hepatocellular ballooning. *Panel* **a** *boxplots* of the reduced scattering coefficient at 1600 nm. *Panel* **b** *boxplots* representing the Mie-slope “b”. For both scattering parameters, no significant differences were found between micro- and macrovesicular steatosis, and hepatocellular ballooning

In total, 104 DRS measurements were performed in 104 hepatic tissue specimens from 52 livers. Examples of optical spectra of macrovesicular, microvesicular steatosis, and hepatocellular ballooning are presented in Fig. 5a. Lipid cells in fatty liver tissue have an absorption peak around 1200 nm [11]. In all the three fat spectra, an inverse peak was observed in the vicinity of 1200 nm. However, discrimination between the three different spectra was not possible due to similar absorption and scattering characteristics of these types of fat around 1200 nm (see also Fig. 6, where boxplots of scattering parameters are presented). Therefore, the DRS system was not able to differentiate between types of fat; only the total hepatic fat fraction could be measured. Figure 5b shows examples of hepatic fat fraction of increasing severity and the corresponding light spectra of the tissue generated with the DRS. Depending on the percentage of hepatic fat fraction; a deeper inverse peak in the vicinity of 1200 nm is related to a higher degree of hepatic fat infiltration. Additionally, in Fig. 6 boxplots are displayed showing the reduced scattering coefficient at 1600 nm and the corresponding Mie-slope “b”. Due to the substantial spread of scattering parameters, both scattering parameters were not significantly different between macrovesicular, microvesicular, and hepatocellular ballooning, indicating that it was not possible to differentiate between the three types of hepatic fat based on this limited data set.

**Fig. 7:**
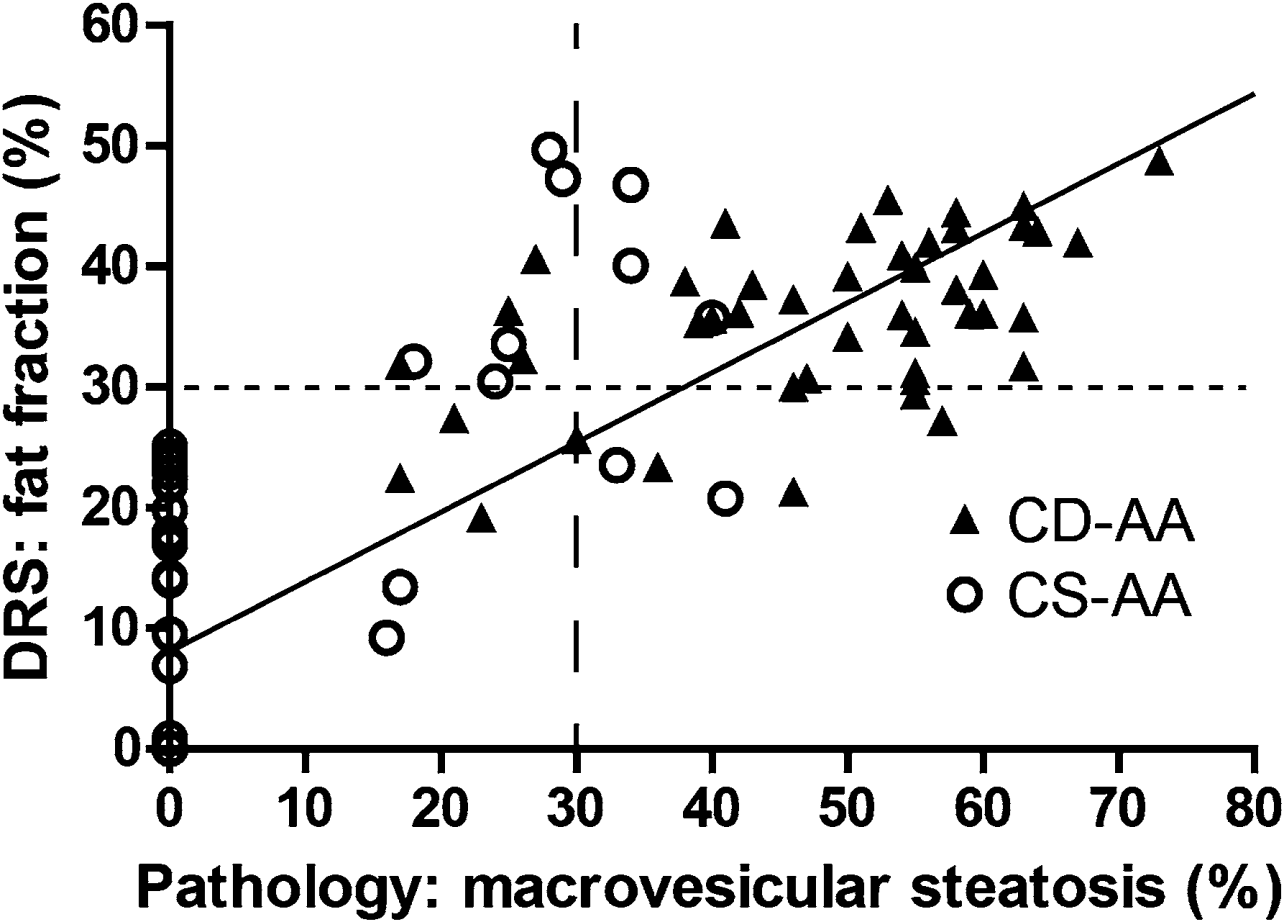
Comparison of pathological evaluation of macrovesicular steatosis and DRS analysis. Comparison between pathological assessment and DRS analyses provides a Spearman’s rank correlation coefficient of 0.761. The *solid line* represents the linear regression line (r^2^) between pathological evaluation and DRS analysis. The *dashed lines* illustrate the quadrants for the sensitivity and specificity calculations above 30 % of macrovesicular steatosis between pathological evaluation and DRS analysis

**Fig. 8:**
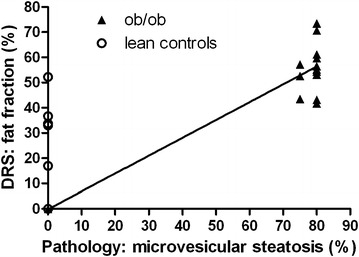
Comparison of pathological analyses of microvesicular steatosis and DRS analysis. Comparison between pathological assessment and DRS analyses provides a Spearman’s rank correlation of 0.747. The *solid line* represents the linear regression line (r^2^) between pathological evaluation and DRS analysis

Figures 7 and 8 shows the correlation coefficients between the pathological evaluation of macrovesicular and microvesicular steatosis and DRS analysis. Because the mouse models differ in patterns of steatosis (macrovesicular versus microvesicular), we were able to calculate the Spearman’s rank correlation coefficients between the two different patterns of steatosis and the total fat fraction analyzed by the DRS. For macrovesicular steatosis the Spearman’s rank correlation coefficient was 0.761 and for microvesicular steatosis 0.747. In addition, the overall sensitivity of DRS to identify macrovesicular and microvesicular steatosis was 100 % (Tables 1, 2). Moreover, the positive predictive value was 80 % for macrovesicular steatosis and 72 % for microvesicular steatosis. Therefore, a high level of agreement was observed between pathological evaluation and DRS analysis in assessment of the hepatic fat content. On the other hand, the specificity of DRS to exclude macro- and microvesicular steatosis was 48 % for macrovesicular and 29 % for microvesicular steatosis, respectively (Tables 1, 2). However, it should be noticed that in the majority of false positive cases (95 %), hepatocellular ballooning was observed in histological analysis. Therefore, the DRS was still measuring fat, despite no macrovesicular or microvesicular steatosis was observed in these cases.

**Table 1:** Accuracy calculations of DRS analysis with respect to macrovesicular steatosis in the CD-AA and CS-AA diet groups

	Result pathology positive for macrovesicular steatosis	Result pathology negative for macrovesicular steatosis	
Positive result DRS analyses	57	14	71
Negative result DRS analyses	0	13	13
	57	27	84
Sensitivity	100 %		
Specificity	48 %		
Positive predictive value	80 %		
Negative predictive value	100 %		

The DRS analyses are positive for steatosis when the determined fat fraction value is larger than 5 %

*DRS* diffuse reflectance spectroscopy

**Table 2:** Accuracy calculations of DRS analysis with respect to microvesicular steatosis in the ob/ob and control mice

	Result pathology positive for microvesicular steatosis	Result pathology negative for microvesicular steatosis	
Positive result DRS analyses	13	5	18
Negative result DRS analyses	0	2	2
	13	7	20
Sensitivity	100 %		
Specificity	29 %		
Positive predictive value	72 %		
Negative predictive value	100 %		

The DRS analyses are positive for steatosis when the determined fat fraction value is larger than 5 %

*DRS* diffuse reflectance spectroscopy

As separate analysis, we assessed in the CS-AA and CD-AA diet group whether the DRS is able to differentiate between mild macrovesicular steatosis and either moderate/severe macrovesicular steatosis (Fig. 7; Table 3). Therefore, we changed the cut-off values for sensitivity and specificity calculations to 30 % of macrovesicular steatosis measured by pathological analysis and to 30 % of hepatic fat fraction assessed by the DRS. Interestingly, above 30 % of macrovesicular steatosis, the DRS predicts hepatic fat fraction with a sensitivity of 86 % and specificity of 81 %.

**Table 3:** Accuracy calculations of DRS analysis in the CD-AA and CS-AA diet groups when cut-off values are changed to 30 % of macrovesicular steatosis measured by pathological evaluation and 30 % of hepatic fat fraction assessed by the DRS

	Result pathology positive for moderate/severe macrovesicular steatosis	Result pathology negative for moderate/severe macrovesicular steatosis	
Positive result DRS analyses	36	8	44
Negative result DRS analyses	6	34	40
	42	42	84
Sensitivity	86 %		
Specificity	81 %		
Positive predictive value	82 %		
Negative predictive value	85 %		

The DRS analyses are positive for steatosis when the determined fat fraction value is larger than 5 %

*DRS*, diffuse reflectance spectroscopy

## Discussion

This study demonstrates that DRS quantifies steatosis with high accuracy in two mouse models of hepatic steatosis. Using the DRS for assessment of the total hepatic fat content showed a good correlation with histological evaluation of the total hepatic fat content. In addition, the DRS is able to discriminate between mild and moderate/severe macrovesicular steatosis with a high sensitivity (86 %) and specificity (81 %). Although DRS can qualify steatosis in liver tissue, the DRS was not able to discriminate between micro- and macrovesicular steatosis.

During the clinical donation procedure, it is necessary to distinguish between mild (0–30 %) and moderate/severe macrovesicular (>30 %) graft steatosis. As already described, transplantation of moderate/severe macrovesicular steatotic donor livers is related to poor postoperative outcome, while transplantation with mild macrovesicular steatotic livers is not associated with inferior outcome [2–4]. Our study showed that DRS is able to differentiate between isolated mild macrovesicular steatosis and moderate and/or severe macrovesicular steatosis with high sensitivity and specificity. So far, there are no tools for accurate steatosis measurements available that can be performed in real-time [4]. In comparison to the currently best measuring methods for steatosis; computer tomography (CT), magnetic resonance imaging (MRI), and magnetic resonance spectroscopy (MRS) [21–24], DRS analysis is easier and less time-consuming, which is beneficial in the short time period of donation. Moreover, it is known that surgeon’s accuracy in predicting steatosis is low, especially when macrovesicular steatosis is above 30 % [25, 26]. As consequence, DRS has the potential to assist the surgical team with estimating the hepatic fat content during the transplantation procedure. Therefore, further research should be focused on development of the DRS system in a hand-held optical instrument.

In the current study, DRS analysis was not able to differentiate between micro- and macrovesicular steatosis and hepatocellular ballooning. Although we hypothesized that the DRS could discriminate between the three types of hepatic fat, we did not find significantly differences in the scattering parameters between the three groups. Therefore, the inability to distinguish between hepatic fat content may have consequences when the DRS is applied in clinical situations. First, micro- and macrovesicular steatosis are occasionally simultaneously present in donor livers [26, 27]. However, in most cases where micro- and macrovesicular steatosis are simultaneously present, the amount of microvesicular steatosis is quite minimal compared to the amount of macrovesicular steatosis [26, 27]. We expect therefore that the overestimation of the degree of hepatic fat infiltration will be negligible when livers with micro- and macrovesicular steatosis are measured with the DRS. Secondly, it could also happen that only microvesicular steatosis is present in donor livers. Hence, there is a risk for a false positive result when a liver with only microvesicular steatosis is measured with the DRS. However, the presence of only microvesicular steatosis is uncommon in potential donor livers [26, 27]. Solely microvesicular steatosis is frequently observed in subtypes of acute liver failure such as the syndrome of Reye and acute fatty liver of pregnancy, due to toxin- or drug-induced impairment of the mitochondrial β-oxidation [28]. Consequently, it is unlikely livers with only microvesicular steatosis would be considered for transplantation and consequently would be measured with the DRS.

Another aspect of hepatic fat infiltration is hepatocellular ballooning. In our experiment some mice livers displayed severe degrees of hepatocellular ballooning. Hepatocellular ballooning is an important histological feature of nonalcoholic steatohepatitis (NASH) [16]. Patients with NASH show in most cases elevated serum levels of transaminases and/or alkaline phosphatase [29]. These elevated levels of transaminases and/or alkaline phosphatase will be noted when the potential donor is screened at the intensive care. Moreover, NASH is related to the development of fibrosis. Fibrotic livers are mostly enlarged and stiff. These characteristics will be observed during assessment of the liver during the donation procedure. Therefore, it is likely that livers of most of these donors with NASH are declined early in the donation procedure. As a result, the risk that livers with severe hepatocellular ballooning are measured with the DRS will be minimal.

A limitation of the current study is that we have used mouse livers instead of human donor livers. However, with respect to C57BL6 mice on a choline-deficient diet, it has been shown that these mice are representative for human features of fatty liver disease, in particular for macro- and microvesicular steatosis [30]. Another limitation regarding the DRS is its invasiveness. However, in our earlier clinical study where we performed in 17 patients’ 49 in vivo DRS measurements, no bleeding complications were observed [10]. In addition, the diameter of the optical DRS needle is smaller (0.8 mm) compared to a general biopsy needle used for liver biopsies during surgery (1.6 mm). Therefore, we expect that the bleeding risk in liver tissue after DRS measurements will be negligible.

## Conclusion

In conclusion, the current study demonstrates that DRS can quantify steatosis with good agreement compared to histopathological analysis. In particular, its feature to differentiate accurately and real-time between isolated mild and moderate/severe macrovesicular steatosis may have clinical relevance. Consequently, further research should be focused on the development of the DRS in a hand-held device. The DRS system in a hand-held device can assists the surgical team in more accurate assessment of the hepatic fat content during organ procurement procedures.

## Abbreviations

CD-AA: choline-deficient l-amino acid-defined diet
CS-AA: choline-sufficient l-amino acid-defined diet
CT: computed tomography
DRS: diffuse reflectance spectroscopy
ECD: extended criteria donor
H&E: hematoxylin and eosin
MRI: magnetic resonance imaging
MRS: magnetic resonance spectroscopy
NASH: nonalcoholic steatohepatitis
